# Everything Old Is New Again: Drug Repurposing Approach for Non-Small Cell Lung Cancer Targeting MAPK Signaling Pathway

**DOI:** 10.3389/fonc.2021.741326

**Published:** 2021-10-06

**Authors:** Anisha S. Jain, Ashwini Prasad, Sushma Pradeep, Chandan Dharmashekar, Raghu Ram Achar, Ekaterina Silina, Victor Stupin, Raghavendra G. Amachawadi, Shashanka K. Prasad, R Pruthvish, Asad Syed, Chandan Shivamallu, Shiva Prasad Kollur

**Affiliations:** ^1^ Department of Microbiology, School of Life Sciences, JSS Academy of Higher Education and Research, Mysuru, India; ^2^ Department of Biotechnology and Bioinformatics, School of Life Sciences, JSS Academy of Higher Education and Research, Mysuru, India; ^3^ Division of Biochemistry, School of Life Sciences, JSS Academy of Higher Education and Research, Mysuru, India; ^4^ Department of Human Pathology, I.M. Sechenov First Moscow State Medical University (Sechenov University), Moscow, Russia; ^5^ Department of Hospital Surgery, N.I. Pirogov Russian National Research Medical University (RNRMU), Moscow, Russia; ^6^ Department of Clinical Sciences, College of Veterinary Medicine, Kansas State University, Manhattan, KS, United States; ^7^ Department of Biotechnology, Acharya Institute of Technology, Bengaluru, India; ^8^ Department of Botany and Microbiology, College of Science, King Saud University, Riyadh, Saudi Arabia; ^9^ Department of Sciences, Amrita School of Arts and Sciences, Amrita Vishwa Vidyapeetham, Mysuru, India

**Keywords:** non-small cell lung cancer, drug repurposing/repositioning, MAPK, targeted therapy, inhibitors

## Abstract

Non-small cell lung cancer (NSCLC) is a prominent subtype of lung carcinoma that accounts for the majority of cancer-related deaths globally, and it is responsible for about 80% to 85% of lung cancers. Mitogen-Activated Protein Kinase (MAPK) signaling pathways are a vital aspect of NSCLC, and have aided in the advancement of therapies for this carcinoma. Targeting the Ras/Raf/MEK/ERK pathway is a promising and alternative method in NSCLC treatment, which is highlighted in this review. The introduction of targeted medicines has revolutionized the treatment of patients with this carcinoma. When combined with current systems biology-driven stratagems, repurposing non-cancer drugs into new therapeutic niches presents a cost-effective and efficient technique with enhancing outcomes for discovering novel pharmacological activity. This article highlights the successful cutting-edge techniques while focusing on NSCLC targeted therapies. The ultimate challenge will be integrating these repurposed drugs into the therapeutic regimen of patients affected with NSCLC to potentially increase lung cancer cure rates.

## Introduction

This review is emphasized on MAPK signaling, its role in tumor progression, and NSCLC targeted therapy. We started with MAPK mutations, and then moved on to drugs that have been reported to target NSCLC. Finally, the review looks into MAPK in NSCLC, with a focus on drug repurposing therapy. The key phrases ‘MAPK,’ ‘drug re-purposing,’ and ‘non-small cell lung cancer’ were searched in PubMed, Google Scholar, ScienceDirect, Nature and NIH National Cancer Institute to find articles from high-quality journals published in the last decade (2011-2021). Articles were checked for uniqueness of subject matter and relevance to the subject area. The search engines resulted in over 956 articles, which were further scrutinized based on certain required criteria. Articles were restricted to the English language only, and a total of 115 articles were selected suitable for the current review.

NSCLC affects 8 of every 10 people with lung cancer, which is caused by a build-up of damaged cells. For several years, this damage can grow, multiply, and spread unchecked. The Ras/Raf/MEK/ERK regulates a number of biological functions, including proliferation and apoptosis. Recent research has discovered that this pathway is also important in regulating cellular senescence (1). Mutations that activate this signaling pathway have been reported in a vast number of human cancers, particularly lung adenocarcinoma, where they are appear to be significant drivers. As a result of these findings, small compounds targeting these kinases have been developed (2). Drug repurposing is an approach for discovering new uses for authorized or investigational medications that aren’t related to their original medical indication. Researchers are increasingly adopting this technique to address the problem of drug shortages in the search for novel cancer medicines. The pharmacokinetic, pharmacodynamic, and toxicological characteristics of drugs have previously been established in preclinical and Phase I research, which is a major advantage of this strategy (3). In the present review, we primarily focus on the anticancer activity of existing drugs that were not initially designed for cancer therapy that can target the key mutations of Ras/Raf/MEK/ERK signaling pathway of NSCLC.

## Non-Small Cell Lung Cancer

The cancer disease affects one out of every six people on the planet, which is greater than malaria, HIV/AIDS, and tuberculosis combined. Globally, 17 million new cancer cases and 9.5 million cancer deaths were estimated in 2018 (4). Lung cancer is the second most often diagnosed cancer and the leading cause of cancer death, with a projected 2.2 million new cases and 1.8 million fatalities in 2020. This accounts for around one in every ten (11.4%) cancer diagnoses and one in every five (18.0%) fatalities. It is the most prevalent cancer in males and the leading cause of mortality. In women, it is the third most common cancer and the second greatest cause of cancer death, after breast and colorectal cancer (5).

**Figure 1:**
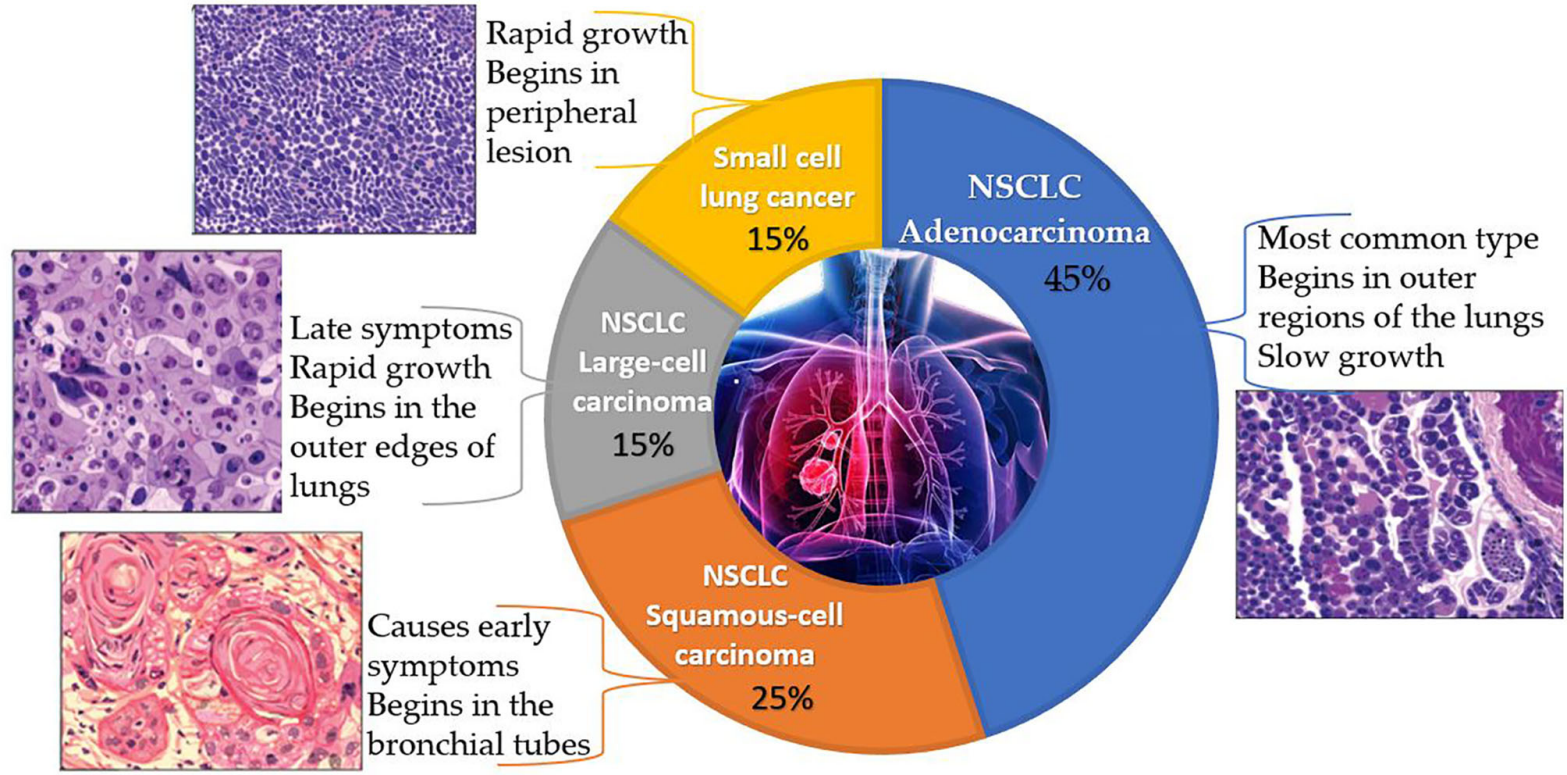
A pie chart representing the classification of lung cancer along with the characteristics, origin and histopathology of each type. (The above histopathology pictures were retrieved from https://www.lungevity.org/).

Lung cancer is a disease that develops when cells in the lungs proliferate and spread uncontrollably. NSCLC and small cell lung cancer (SCLC) are the two main forms, represented in **Figure 1**. NSCLC accounts for 80 to 85% of lung cancer cases, with the remainder being SCLC. Adenocarcinoma, squamous cell carcinoma, and large cell carcinoma are the three primary subtypes of NSCLC. Of all the types, adenocarcinoma is the most common form that makes up 40% of all lung cancers. Adenocarcinoma begins in the cells of the glands on the outside of the lungs and is most common in non-smokers, women, and people under 45 years old. Squamous cell carcinomas account for 25–30% of all lung cancers and are attributed largely to people with a history of smoking. Men are more likely than women to develop this subtype of NSCLC (6). A majority of this carcinoma begins centrally, in the lung’s larger bronchi. Large cell carcinoma accounts for 10-15% of cases, which is the rarest of all lung cancers. This subtype of carcinoma develops rapidly and is often undetected until it has metastasized to other parts of the body (7).

**Figure 2:**
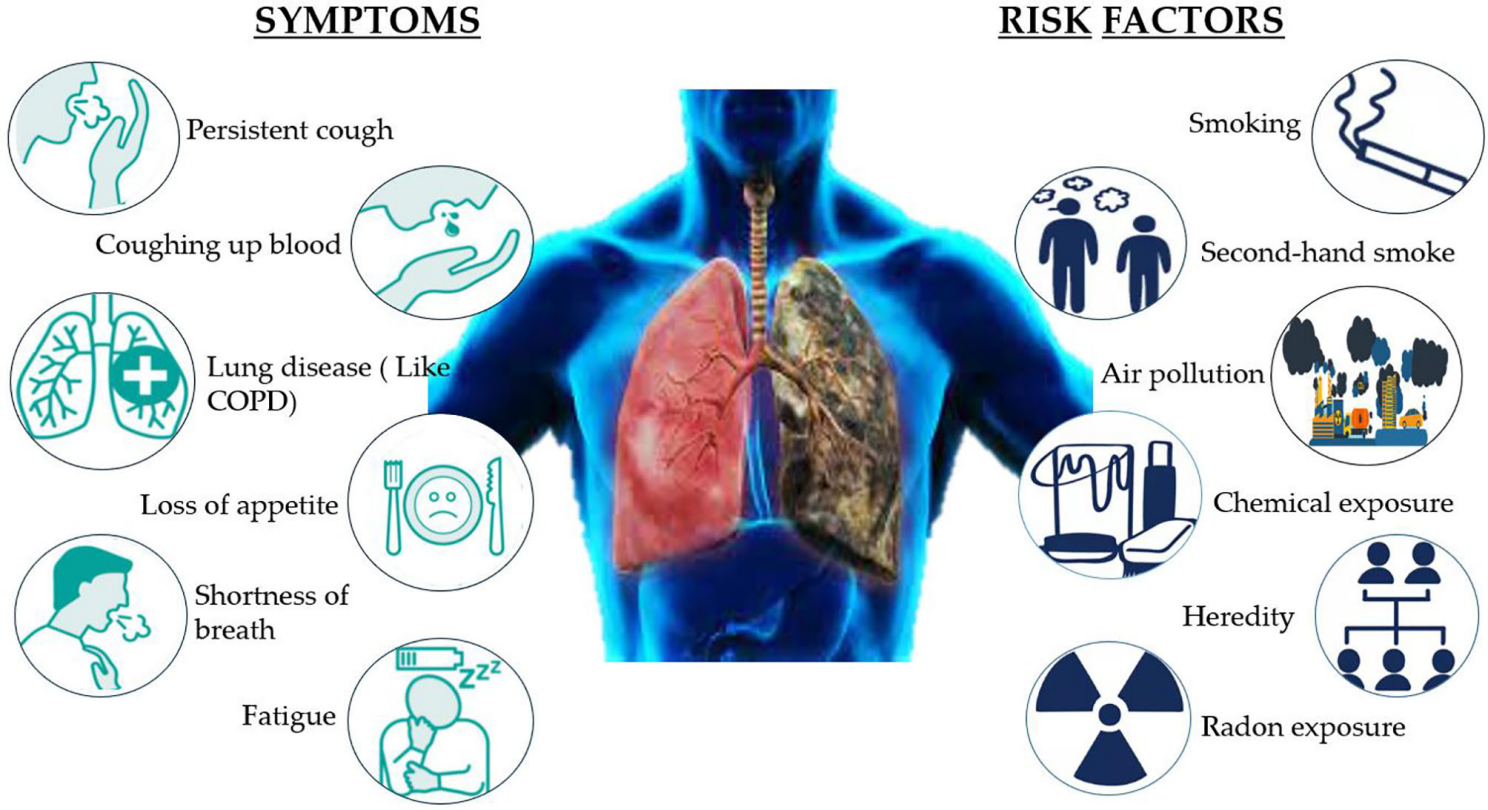
A few symptoms and risk factors causing NSCLC in humans.

NSCLC affects 8 of every 10 people with lung cancer, which is caused by a build-up of damaged cells. For several years, this damage can grow, multiply, and spread unchecked. There are certain risk factors of NSCLC that can be avoided, such as smoking, being exposed to second-hand smoke, or exposure to radon, asbestos, uranium, and other radioactive materials and chemicals like arsenic, coal products, etc. A few risk factors cannot be altered, such as air pollution, heredity, and prior radiation therapy to lungs. The symptoms of NSCLC are not specific, but possible symptoms include persistent cough, rust-colored spit, coughing up blood, shortness of breath, hoarse voice, persistent lung infection, fatigue, and loss of appetite. Eating a healthy diet, avoiding smoking cigarettes, and exposure to harmful chemicals can reduce the risk of lung cancer (**Figure 2**) **(** 8).

**Figure 3:**
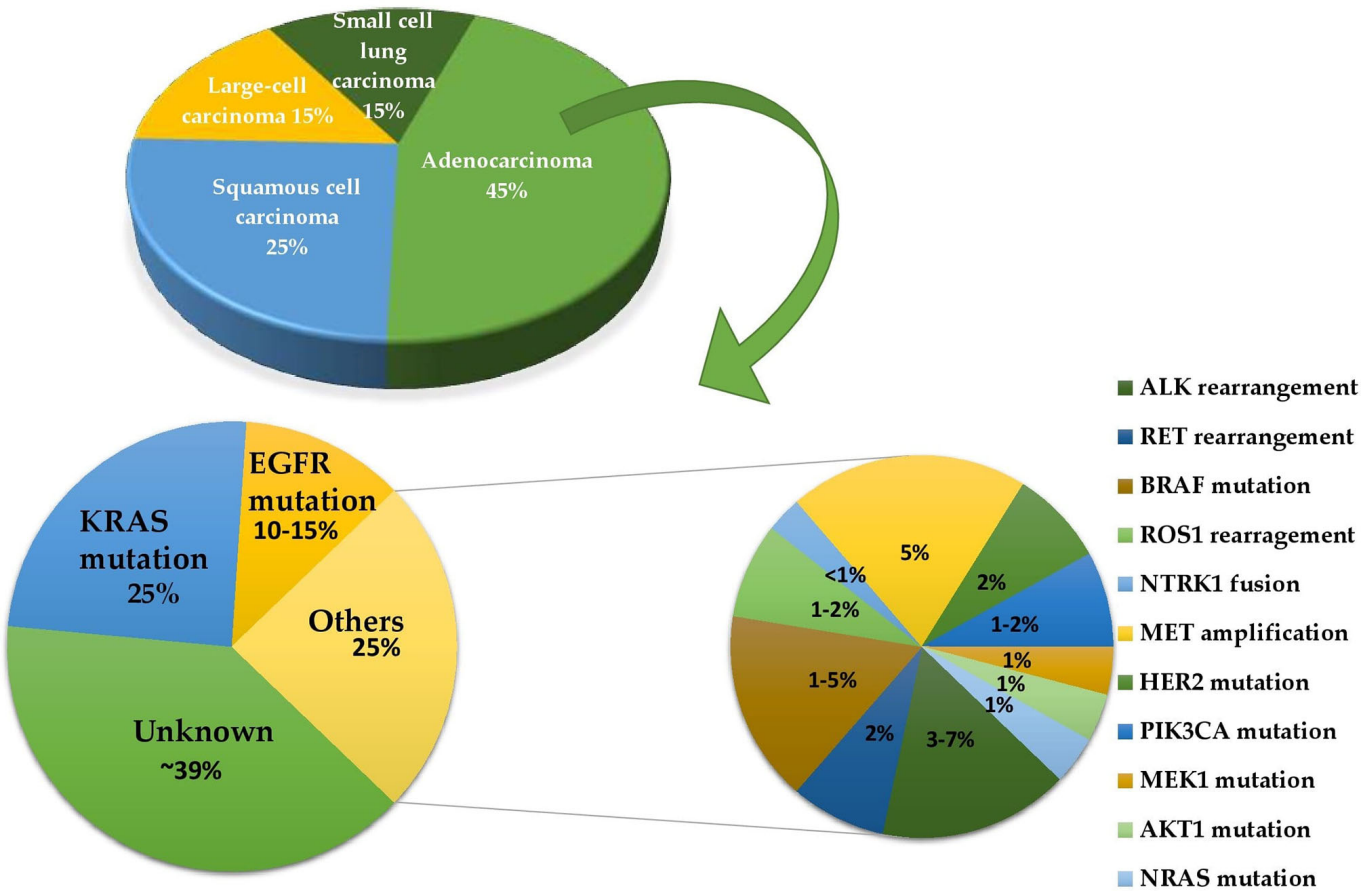
A pie of pie chart determining the frequencies of different driver mutations in lung adenocarcinoma.

The most important treatment option for NSCLC is targeted therapy, and other basic treatment options include radiation therapy, surgery, chemotherapy, and immunotherapy. This review is based on the targeted therapy *via* a drug repurposing approach. Targeted therapies focus on a particular protein that is malfunctioning and causing cancer to grow. It targets cancer cells’ specific mutations that distinguish them from healthy cells (**Figure 3**). For this therapy, only patients whose malignancies test positive for the drug target are given the drugs. Monoclonal antibodies and small-molecule inhibitors are the two forms of targeted therapeutics. Monoclonal antibodies are designed to target alterations on the surface of cancer cells. These drugs include small-molecule inhibitors that attack cancer cells’ internal changes, and are administered using an intravenous line. These drugs can also be prescribed as pills once or twice a day. Targeted therapy is usually given for patients with stage IV NSCLC, where cancer has spread to other organs (9, 10). The discovery of mutations in lung cancer has contributed to the advancement of molecularly targeted therapy to help affected patients survive longer. Mutations in genes that encode elements of the EGFR, PI3K, and downstream MAPK signaling pathways can now be used to classify subtypes. This review primarily emphasizes the mutations of the MAPK pathway. These mutations can be used to define drug sensitivity, as well as primary or acquired resistance to kinase inhibitors (11).

## Mitogen-Activated Protein Kinase (MAPK) Pathway

**Figure 4:**
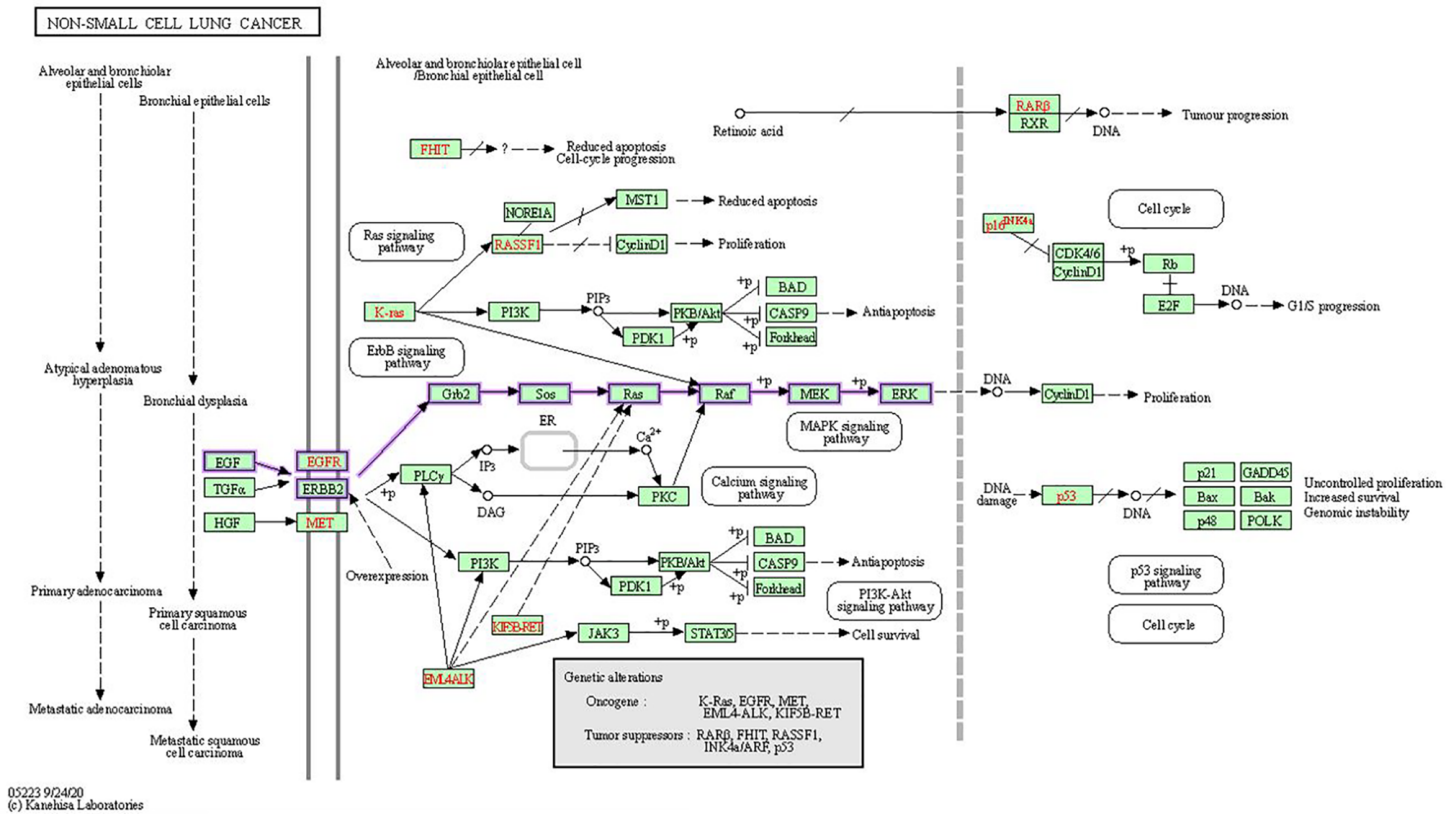
In NSCLC, oncogenes including EGFR, KRAS, and EML4-ALK are activated, while tumor-suppressor genes including RAR-beta, RASSF1, p16INK4a and p53 are inactivated. In the KEGG MAPK signaling network, the Ras/Raf/MEK/ERK signaling pathway is emphasized (13).

In human cancer, the MAPK signaling pathway has a dominant role in several cellular functions, including cell survival, differentiation, proliferation, metastasis, and apoptosis. Overexpression of its elements, which are known as oncogenes, results in a large variety of tumors (12). MAPK pathways are three-kinase cascades where the most upstream kinase (MAPKKK) responds to various extracellular and intracellular signals, then directly phosphorylates the middle kinase (MAPKK) (**Figure 4**). MAPKK phosphorylates and activates a MAPK, which usually has a large number of substrates that carry out complex cell fate decisions in response to the input signal. MAPKs are serine/threonine protein kinases that belong to a broad family (14). MAPK14 (also known as p38-α), JNK (also known as stress-activated protein kinases (SAPK)) and extracellular-signal-regulated kinase MAPK (ERK MAPK) (also known as Ras/Raf/MEK/ERK) are the three most common subfamilies of MAPK. The ERK MAPK is primarily engaged in lung cell death, pathogenesis, development, and carcinogenic activity. Kinases concerned in this cascade include RTKs, Ras, Raf, MEK, and ERK. The development of NSCLC is primarily influenced by four main mechanisms.

### Receptor Tyrosine Kinases (RTKS)

RTKs are kinases that are upstream of the signaling and are intricated in the Ras/Raf/MEK/ERK pathway. Growth factors activate the signal transduction cascade by binding and activating RTK transmembrane glycoproteins, followed by a signal transmission *via* cytosolic intermediates, and then transcription/translation regulation of effector genes occurs (15). This family comprises the epidermal growth factor receptor (EGFR) and the fibroblast growth factor receptor (FGFR) (16). The binding of a ligand/GF, such as EGF, stimulates EGFR, which is then activated by an intracellular tyrosine kinase domain. This causes it to autophosphorylate, resulting in EGFR overexpression and increased intracellular pathway activity. As a result, non-small lung cells engage in atypical cellular activity. Around 40 to 89% of NSCLC patients have EGFR deregulation (12). SHP2 and GRB2 control the activation of SOS proteins, first to SOSs, then to GDP and GTP, and finally GTP activates RAS. As a result, SHP2 and/or GRB2 inhibitors are successful in disrupting RAS-GTP loading in tumors with mutations of MAPK dependent kinases (17). The three key mechanisms that lead to EGFR activation include increased EGFR expression on malignant cells, increased ligand output by malignant cells, and triggering mutations of EGFR within malignant cells. EGFR was regarded to be a promising translational therapeutic target because it is overexpressed in up to 40% to 80% of NSCLC patients. However, it was later discovered that activating mutations, rather than EGFR overexpression, was the primary therapeutic target (18).

### RAS

Ras is a small GTPase that regulates upstream and downstream protein interactions, and GTPase hydrolyses GTP into GDP. RAS proteins are bound to the plasma membrane’s internal surface and function as binary switch kinases (19). Ras is enabled by members of the RTK family, such as EGFR (16). The protein is inactive when bound to GDP, but when bound to GTP, it becomes active. This results in a conformational shift that allows downstream effectors to bind to and activate Ras, which triggers signaling cascades. A mutation in the Ras gene is detected in roughly 30% of human solid tumors, according to data from cancer mutation databases (20). HRAS, KRAS, and NRAS are the three subfamilies of the Ras gene. By binding to certain effectors (MAPK), these proteins encode membrane-bound 21-kD GTP-binding proteins that in-fluence cell responses, such as metastasis and apoptosis. This can result in a Ras mutation with aberrant GTPase action, which could lead to NSCLC development. Oncogenic mutations in the RAS family of genes result in amino acid changes at three key residues—Gly12, Gly13, and Gln61—each of which precludes the hydrolysis of bound GTP to GDP, leading to an active protein. Approximately 20–30% of lung adenocarcinomas are caused by a mutation in the Ras gene (21). KRAS is chiefly mutated in lung, colorectal, and pancreatic cancers. NRAS activation is common in lymphoid/hematopoietic cancers and melanomas. HRAS is the least common cancer-associated Ras’s isoform, and it is found mostly in cancers of the urinary tract, such as bladder cancer. The most common mutation sites in all three canonical Ras proteins are codons 12, 13 or 61 (22).

KRAS activating mutations that contribute to constitutive signaling are found more frequently in adenocarcinoma (30%) and less frequently in squamous cell carcinoma (7%) (18, 23). KRAS mutations are most typically identified in the tumors of smokers (particularly heavy smokers), with non or light smokers accounting for just 5–10% of KRAS-mutant lung cancers. The majority of KRAS mutations in NSCLC contain codons 12 (90%) or 13 (>80%) and are normally linked to a history of tobacco use (24). The KRAS-G12C mutation is the most prevalent codon variation, accounting for roughly 39% of all KRAS-mutant NSCLCs. Other prevalent mutations are KRAS-G12V (18–21%) and KRAS-G12D (17–18%). The KRAS mutations and codon variants differ between smokers and non-smokers. Former or current smokers are more likely to have transversion mutations (G > C or G > T), whereas non-smokers are more likely to have transfer mutations (G > A) (25, 26).

### RAF

Raf is a downstream effector of Ras; hence, it must interact with an active Ras. A-Raf, B-Raf, and C-Raf are the three major variants of the Raf family, all of which are serine/threonine kinases that activate MEK and ERK1/2 to promote pathway progression. RAF1 (also known as CRAF) mutations are less prevalent (about 2%) although multiple studies have found elevated RAF1 expression in a range of primary human cancers (27). Of all the variants, BRAF mutations are frequent (12). Approximately 10% of mutations in BRAF contribute to the alteration of the Ras-Raf-MEK-ERK pathway, which leads to 40% of all human cancers. In 2-4% of NSCLC patients, these mutations have been discovered. The two types of Ras-independent BRAF mutants include class I mutants that behave as monomers and class II mutants that function as dimers. V600E is a class I BRAF mutant with the most widespread mutations (50%), and its constituent activation leads to MAPK hyperactivation (28). Only tumors with V600 mutations dis-play consistent clinical responses (17). In a negative feedback loop, MEK phosphorylation is caused by BRAF activation, which limits BRAF activity. BRAF inhibition has been linked to MAPK reactivation, which is thought to be mediated by EGFR (16).

### Mitogen-Activated Protein Kinase Kinase (MEK)

MEK proteins are dual-specificity Tyr/Thr protein kinases that phosphorylate serine/threonine and residues of tyrosine in ERK1 and ERK2 (29). MEK proteins are encoded by seven distinct genes, the most important of which are MEK1 and MEK2. Raf isoforms activate MEK (via phosphorylation), and MEK’s downstream target is ERK (16, 30). Approximately 2% of MEK mutations occur in NSCLC patients. Since the Raf-MEK-ERK pathway is normally activated, it contributes significantly to tumor cell proliferation and survival. As a result, MEK1/2 inhibitors have the most antitumor effects in tumors harboring Ras or BRAF activating mutations (18).

### Extracellular Signal-Regulated Kinase (ERK)

ERK is another serine/threonine-protein kinase. ERK, like MEK, has two subunits: ERK1 and ERK2, which are activated by phosphorylation (31). When several kinases act on MEK, it directly cooperates with ERKs *via* its N-terminal domain, catalyzing the bispecific phosphorylation of Thr and Tyr residues. MEK activates ERK, while also an-choring it in the cytoplasm. If the signaling pathway is dormant, ERK is found in the cytoplasm. Activated ERKs are translocated to the nucleus when a signal induces the phosphorylation and dimerization of ERK (32). MEK and ERK mutations are uncommon. A blockade of ERK could cause patients to overcome or postpone resistance to inhibitors of upstream kinases like MEK and BRAF, which could benefit a larger range of patients suffering from cancer (16, 19).

## Inhibitors of RAS/RAF/MEK/ERK (MAPK) Pathway

The MAPK pathway (Ras, Raf, and MEK), one of the most downregulated path-ways in cancer, has recently been discovered to be a feasible target for innovative cancer therapy. Appropriate drugs ought to be target-specific and potentially less harmful than traditional cancer treatment (12). EGFR inhibitors effectively bind to EGFR, inhibiting EGFR overexpression and proliferation in NSCLC by reducing the binding of alternative ligands. Both gefitinib (33) and erlotinib (34) are FDA-approved EGFR inhibitors that could potentially be utilized to treat NSCLC (35). Trametinib and cobimetinib, two MEK inhibitors, have been approved by the EMA and FDA (35). In a pre-clinical study, trametinib showed tumor growth inhibition of 92% at 5.0 mg/kg and 87% at 2.5 mg/kg, in an A549 (KRAS mutant cell line) xenograft model (36). Cobimetinib is a powerful and extremely selective MEK inhibitor that has shown extensive activity in xenograft models using KRAS- and BRAF-mutated cell lines *in vivo* (37). The FDA has approved trametinib and dabrafenib as a breakthrough designation for BRAF-mutant NSCLC in 2015, and the combination was authorized in June 2017. A new therapy option has been obtained by combining MEK and BRAF inhibitors (38).

KRAS is the only protein in this pathway for which there are no medicines that target its function directly. Oncogenic Ras mutants have been termed “undruggable” for decades, because of their high affinity for GTP and absence of a suitable binding site for small molecule inhibitors to bind (39). A covalent small molecule that docks in the switch II pocket and cross-links with Cys12 can be used to target KRAS G12C, ac-cording to the Shokat lab at UCSF (40). These results initiated a rush to produce KRAS-G12C-targeting medicines for therapeutic usage. The first drugs to target the KRAS G12C mutation in NSCLC were AMG510 and MRTX125 (41, 42). However, more research into these drugs is still needed. Many researchers are attempting to tar-get Ras signaling downstream effectors as an alternative, such as MEK inhibition. To treat KRAS-driven cancers, a single or combinatorial therapeutic strategy could be used.

**Figure 5:**
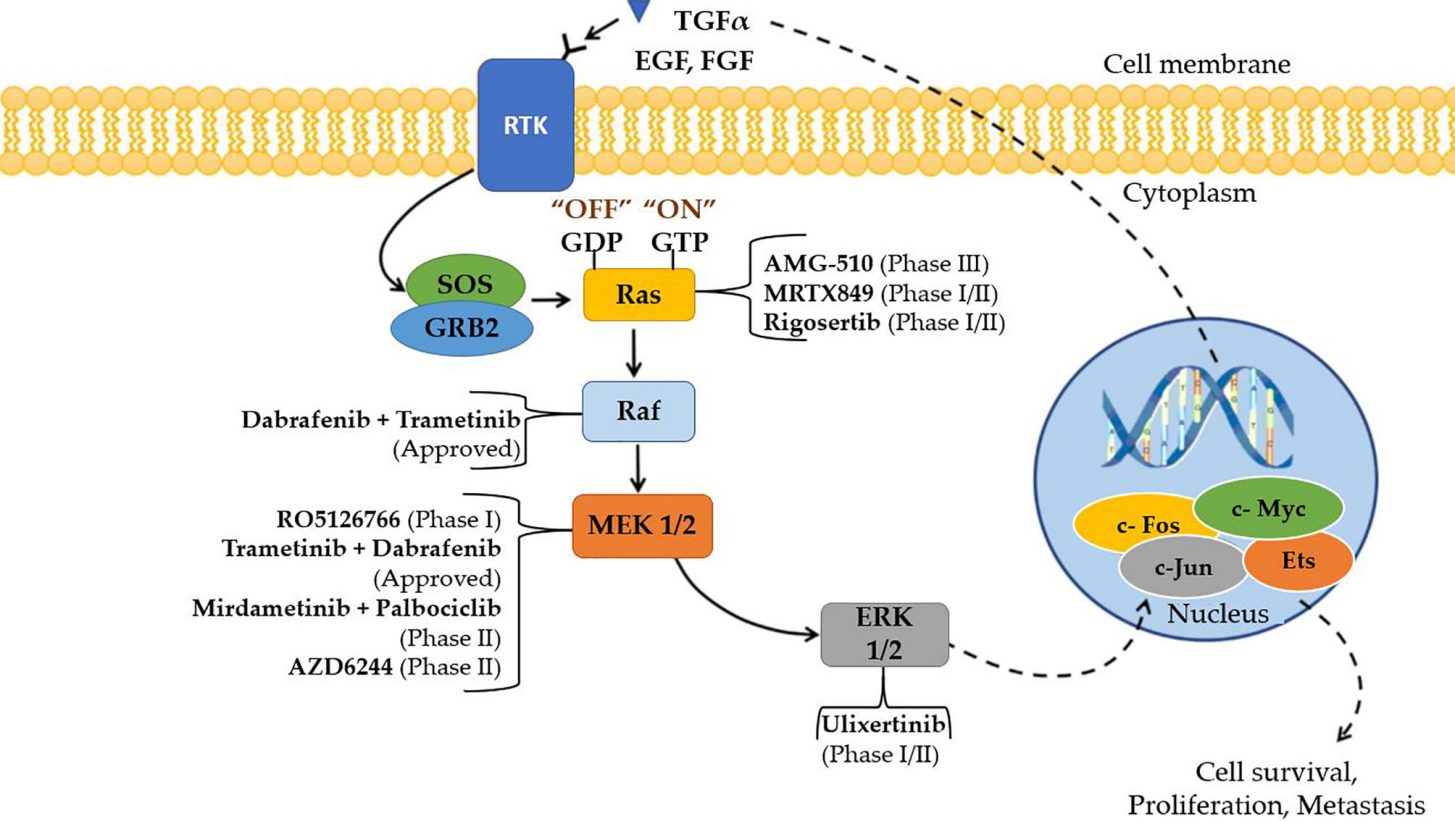
Small molecule inhibitors that are approved and are in clinical trials targeting Ras/Raf/MEK/ERK (MAPK) cascade for the treatment of NSCLC (48, 49).

Among the components of this signaling cascade, Raf is a significant direct effector of Ras mutants, and a major target of carcinogenic mutations. RAF has long been considered a promising target for cancer therapy research because it is the first kinase in this pathway (43). The first-generation Raf inhibitors, including vemurafenib (44), dabrafenib (45) and encorafenib (46), were developed and used to treat BRAF(V600E)-positive malignancies as single treatments or in combination with MEK inhibitors. In the early stages of treatment, these drugs had promising efficacy, but it was gradually destroyed by drug resistance. This pathway can be activated by cancer cells in response to drug therapy in two ways: 1. alternate splicing of BRAF(V600E) to produce variants with shortened N-termini, which improves BRAF(V600E) homodimerization and declines drug affinity; and 2. upregulating the cellular level of active Ras, which prompts to paradoxical initiation of ERK signaling. Other small molecule inhibitors, such as selumetinib and binimetinib, as well as a slew of others, are in the preclinical or early clinical stages of development (47). **Figure 5** explains the Ras/Raf/MEK/ERK Pathway diagrammatically and the specific inhibitors of each mutation of the pathway are specified. Cell surface molecules and protein kinases are still the most popular targets for anticancer drug development. A key focus for future MAPK drug development should be on targets that aren’t traditionally thought of as attractive or “druggable,” but are crucial modulators of MAPK function (50).

## Immune Checkpoint Inhibitors

Tumor cells upregulate immune checkpoint molecules in the tumor microenvironment to suppress the immune system’s anti-cancer response. As a result, blocking immunological checkpoints with selective monoclonal antibodies is predicted to inverse the inhibition of tumor-specific immune cells like T cells and natural killer cells. Checkpoint blockage, in particular, has been demonstrated to produce long-term effects; nonetheless, the response rate is lesser than that of targeted therapy. Currently licensed checkpoint inhibitors do not produce long-term therapeutic responses in over 80% of cancer patients when administered as monotherapy. As a result, one of checkpoint therapy’s limitations is the scarcity of activated T cells that can respond to it. Furthermore, tumors that show an early response to checkpoint therapy may grow resistant to it, reducing therapeutic efficacy even further (51). Immune checkpoint blockades (ICB) (especially anti-PD-1/L1 treatments) combined with MEK and BRAF inhibitors are presently being studied in clinical trials. To establish the possible toxicity of any specific drug when used in combination therapy, long-term research is required. Multiple clinical trials have investigated at the blend of BRAF inhibitors with anti-CTLA-4 antibodies. In several studies, however, significant immune-related side effects were the main source of concern. In trials involving the combination of vemurafenib and ipilimumab, liver damage and severe cutaneous side effects were seen. Patients who got the triple combination of dabrafenib, trametinib, and ipilimumab developed severe colitis (NCT01767454). The combination of trametinib, durvalumab and dabrafenib showed encouraging disease response rates and tolerable safety profiles in a phase 1 trial (52). To define the sequencing and scheduling of the combination, swift growth of resistance to BRAF/MEK inhibitors, as well as their dynamic effects on the tumour microenvironment must be taken into account. When it comes to the combination of targeted therapies and ICB, there are certain obstacles that need to be addressed in the future (53).

## Resistance to Targeted Therapy in NSCLC

Despite the advances made in cancer treatment over the last few decades, resistance to traditional anticancer agents and/or new targeted drugs remains a key issue in the field of oncology. Unfortunately, the initial therapeutic response to targeted kinase inhibitors is usually always transient, since these medications develop acquired resistance. In 40% of all human malignancies, the MAPK pathway is disrupted, owing to mutations in RAS (30%) and BRAF (10%) (54). MEK inhibitors were the initial drugs to be discovered, but despite their high selectivity and potency, they were mainly unsuccessful in clinical trials. This failure is due to the pathway’s negative feedback amplifier feature, which autocorrects perturbations to the amplifier, i.e., MEK, in order to maintain ERK signaling (55). Patients acquire drug resistance after 10–14 months of first-generation EGFR-TKI therapy, according to previous research (56). Amplification of MET, T790M (TK domain mutation) and mutation in RAS have all been identified as drug resistance mechanisms in first-generation EGFR-tyrosine kinase inhibitor in NSCLC (57). The most prevalent acquired resistance mutation in patients with NSCLC is the TK domain mutation (T790M) (58). Osimertinib, a third-generation TKI, recently improved outcomes in patients with this novel mutation (59). Other molecular resistance pathways have been identified, but more knowledge is needed to better understand and overcome resistance to EGFR-TKIs in the 40–50% of patients who do not have the T790M mutation. Amplification of the MET gene, regardless of T790M mutation status, is the second most prevalent route of acquired resistance, affecting roughly 5–20% of NSCLC patients during EGFR-TKI treatment. The emergence of mutations in KRAS, TP53, and CDKN2A has been hypothesized as a resistance mechanism to the BRAF inhibitor, dabrafenib in the clinics for BRAF mutated lung adenocarcinoma (60). In NSCLC, clear evidence of resistance to BRAF and MEK inhibitors are yet to be reported (61).Because cancers are virtually usually multiclonal and genetically heterogeneous, combination therapy is widely recommended. Single-drug therapeutic techniques are most likely to fail due to drug resistance, as the therapy kills sensitive cancer cells while allowing resistant cancer cells to live and multiply. Combination therapy, on the other hand, is more likely to target many driver genes at the same time, suppressing more clones in a tumour but also making future cancer mutations resistant to multi-drug treatment. Simultaneous multi-targeting, like previous advances in successful target drug therapy, will be more effective in overcoming drug resistance, improving anticancer efficacy and extending patients’ survival. Blocking the energy source of tumour cells is one technique for overcoming resistance. Tumors can circumvent any mechanism, but they cannot avoid the need for energy to fuel their growth, proliferation, and other activities including drug resistance and cell migration. However, therapeutic effects are inextricably linked to the composition/unique resistance profile of malignancies, as well as the toxicity tolerance of patients, making therapeutic outcomes difficult to forecast. Fighting drug resistance appears to be an ongoing game because cancer cells can always discover new strategies to get around present treatment (62).

## Drug Repurposing

Drug repurposing, also called repositioning, is an approach to find novel applications for used medications or the ones that failed due to a lack of efficacy rather than developing new molecules (*de novo* drug development). When compared to the traditional drug development process, repurposing has many benefits, the two most important of which are a reduced risk of failure due to safety and a shorter development time. From concept to market, the total cost of producing a new drug is projected to be $1.8 to 2.6 billion. Additionally, the entire process will take 10–15 years. Approval of drugs *via* the repurposing route is expected to take 3–12 years and cost $40–80 million, which is considerably less than the traditional method of drug development (63, 64). Furthermore, the approval rate of drugs developed through the repurposing route is projected to be 30%, compared to 10% for drugs developed through the traditional route. The safety and adverse reactions of approved drug libraries, along with the secondary targets of FDA-approved drugs, can be effortlessly screened using genomics, metabolomics, systems biology, knowledge about signal transduction pathways, and by using deep-data mining techniques (65).

**Figure 6:**
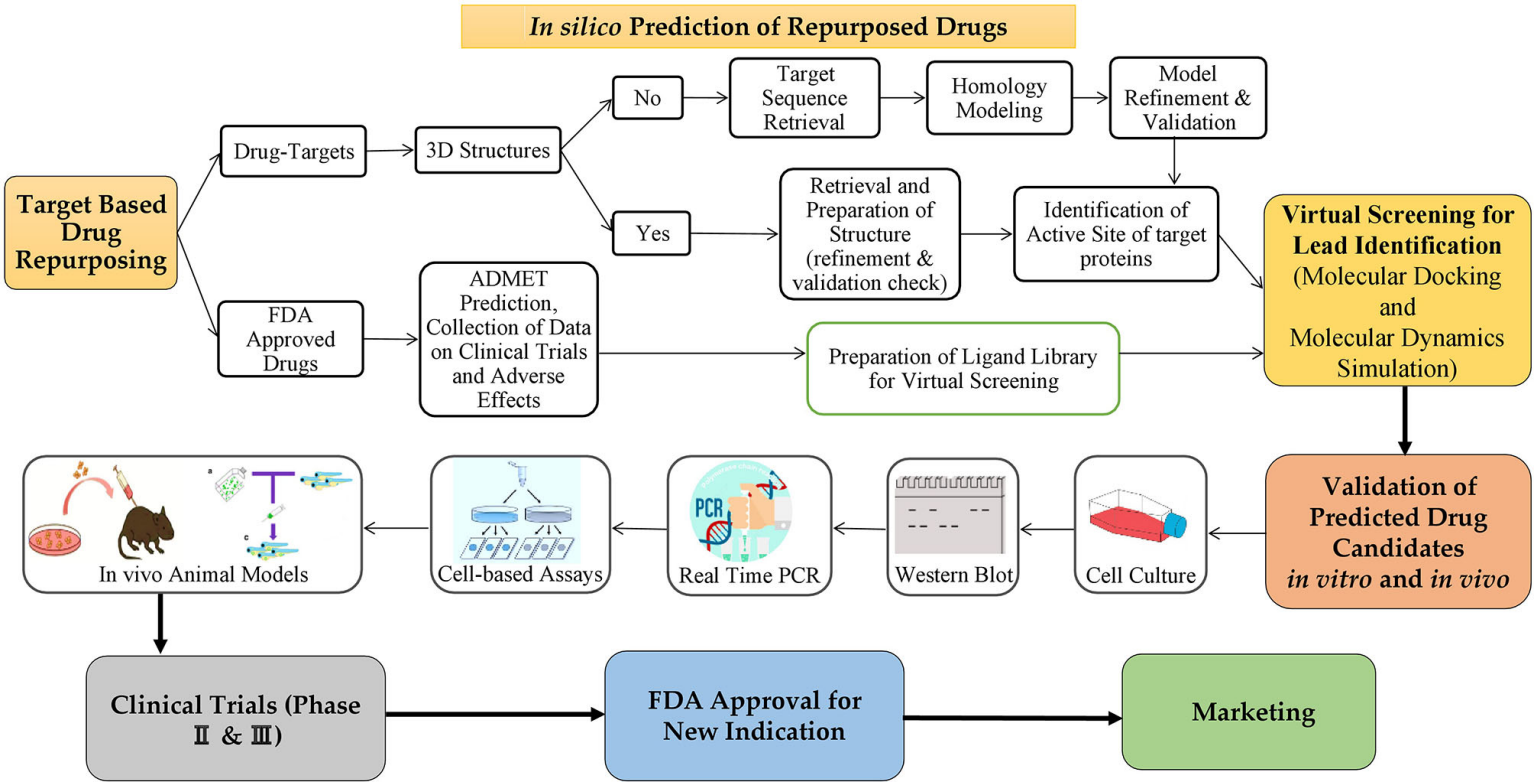
The process of repurposing a drug for a new indication. Target based drug repurposing: Potent drug candidates targeting NSCLC can be predicted through *in silico* target-based drug repurposing approach based on several databases, tools and software. Then anticancer properties of predicted drugs can be validated *in vitro via* several cell-based assays for cancer followed by *in vivo* animal models. Further validation is done in clinical trials (phases 2 & 3 only) and then the drugs to be repurposed can be approved by FDA for clinical usage on the market.

By combining statistical modelling, clinical and pharmacological evidence, and experimental trials, computer-assisted drug repurposing has the potential to quickly assess the majority of features based on safety and efficacy (**Figure 6**). Many analytical methods based on diverse data formats and approaches have been presented in cancer-related drug repurposing literature. From traditional statistical approaches to cut-ting-edge machine learning techniques, there are a variety of methodologies to choose from (66). *In silico* drug repurposing may further increase the efficacy of personalized targeted cancer therapies. Modern oncology faces several challenges, one of which is to offer personalized and targeted cancer treatments with the aim of reducing drug toxicity and increasing each patient’s response rate (67). **Table 1** shows a list of *in silico* resources used for identifying potential repurposing candidates for NSCLC targeted therapy.

**Table 1:** List of avail1able tools/databases/software for *in silico* drug repurposing approach.

Tools/Databases/Software		Links
**I. Drug Target 3D Structure and Sequence Database**
➢RCSB Protein Data Bank (PDB) ➢GeneCards^®^: The Human Gene Database ➢The Human Protein Atlas ➢Pharos ➢Therapeutic Target Database (TTD)		https://www.rcsb.org/ https://www.genecards.org/ https://www.proteinatlas.org/ https://pharos.nih.gov/ http://db.idrblab.net/ttd/
**II. Protein Modelling**
➢SWISS-MODEL ➢Phyre2 ➢I-TASSER ➢MODELLER		https://swissmodel.expasy.org/ http://www.sbg.bio.ic.ac.uk/phyre2/html/page.cgi?id=index https://zhanglab.ccmb.med.umich.edu/I-TASSER/ https://salilab.org/modeller/
**III. Protein Refinement and Optimization**
➢GalaxyRefine ➢ModRefiner ➢3Drefine		http://galaxy.seoklab.org/cgi%20bin/submit.cgi?type=REFINE https://zhanglab.dcmb.med.umich.edu/ModRefiner/ http://sysbio.rnet.missouri.edu/3Drefine/
**IV. Protein Validation**
➢MolProbity ➢ProSA-web		http://molprobity.biochem.duke.edu/ https://prosa.services.came.sbg.ac.at/prosa.php
**V. Pathway Information**
➢KyotoEncyclopediaof Genes and Genomes (KEGG) ➢Cytoscape ➢Reactome		https://www.genome.jp/kegg/pathway.html https://cytoscape.org/ https://reactome.org/
**VI. Target Binding Site Prediction**
➢GalaxySite ➢COACH ➢CASTp ➢3DligandSite		http://galaxy.seoklab.org/cgi-bin/submit.cgi?type=SITE https://zhanglab.dcmb.med.umich.edu/COACH/ http://sts.bioe.uic.edu/castp/index.html?2pk9 http://www.sbg.bio.ic.ac.uk/~3dligandsite/
**VII. Drug/Small Molecule Databases**
➢DrugBank ➢PubChem ➢Therapeutic Target Database (TTD) ➢ZINC ➢ChEMBL		https://go.drugbank.com/ https://pubchem.ncbi.nlm.nih.gov/ http://db.idrblab.net/ttd/ https://zinc.docking.org/ https://www.ebi.ac.uk/chembl/
**VIII. FDA Label Information**
➢Pharmacognetics knowledge base (PharmaGKB) ➢FDA Label Search ➢DailyMed ➢ClinicalTrials.gov		https://www.pharmgkb.org/ https://labels.fda.gov/ https://dailymed.nlm.nih.gov/dailymed/ https://clinicaltrials.gov/ct2/home
**IX. Drug-target Association**
➢Connectivity Map ➢STRING ➢PharmGKB ➢ChemMapper		https://clue.io/repurposing-app https://string-db.org/ https://www.pharmgkb.org/ http://lilab-ecust.cn/chemmapper/
**X. Clinical Trial Information and Adverse Effects**
➢SIDER ➢Drug Side Effects ➢DailyMed ➢ADVERPred ➢SuperDRUG2		http://ffects.embl.de/ https://www.drugs.com/sfx/ https://dailymed.nlm.nih.gov/dailymed/index.cfm http://www.way2drug.com/adverpred/ http://cheminfo.charite.de/superdrug2/index.html
**XI. ADMET Prediction**
➢ADMETlab ➢SwissADME ➢CLC-Pred		http://admet.scbdd.com/ http://www.swissadme.ch/ http://way2drug.com/Cell-line/
**XII. Screening**
➢SwissSimilarity ➢PASSonline ➢ZincPharmer ➢Docking-based Virtual Screening (DOVIS)		http://www.swisssimilarity.ch/ http://way2drug.com/PassOnline/pe.php http://zincpharmer.csb.pitt.edu/ http://bhsai.org/software/
**XIII. Docking Software**
**Free**	**Paid**	
➢AutoDock ➢AutoDock Vina ➢UCSF DOCK ➢SwissDock ➢PyRx	➢GOLD ➢Glide ➢Cdocker	http://autodock.scripps.edu/ http://vina.scripps.edu/ http://dock.compbio.ucsf.edu/ http://www.swissdock.ch/ https://pyrx.sourceforge.io/
		https://www.ccdc.cam.ac.uk/solutions/csd-discovery/Components/Gold/ https://www.schrodinger.com/products/glide https://www.3ds.com/products-services/biovia/
**XIV. Analysis of Protein-Ligand Interactions**
➢UCSF Chimera ➢Maestro ➢Discovery Studio Visualizer ➢PyMol ➢Protein-Ligand Interaction Profiler		https://www.cgl.ucsf.edu/chimera/ https://www.schrodinger.com/products/maestro https://discover.3ds.com/discovery-studio-visualizer-download https://pymol.org/2/ https://plip-tool.biotec.tu-dresden.de/plip-web/plip/index
**XV. Molecular Simulation and Dynamics**
**Free**	**Paid**	
➢GROMACS ➢NAMD ➢Simlab WEBGRO	➢BIOVIA Discovery Studio Simulations ➢Desmond	http://www.gromacs.org/ http://www.ks.uiuc.edu/Research/namd/ https://simlab.uams.edu/ProteinWithLigand/protein_with_ligand.html
		https://www.3ds.com/products-services/biovia/products/molecular-modeling-simulation/biovia-discovery-studio/simulations/ https://www.schrodinger.com/products/desmond
**XVI. Cancer-related Database and Tools**
➢The Cancer Genome Atlas (TCGA) ➢DRUGSURV ➢IntOGen ➢Cancer Cell Line Encyclopaedia (CCLE) ➢CellMiner ➢OncoPPi Portal ➢TNMplot ➢canSAR Black ➢The Cancer Therapeutics Response Portal (CTRP) ➢The PRISM drug repurposing resource		https://www.cancer.gov/about-nci/organization/ccg/research/structural-genomics/tcga http://www.bioprofiling.de/cgi-bin/GEO/DRUGSURV/start_CANCER.pl https://www.intogen.org/search https://portals.broadinstitute.org/ccle https://discover.nci.nih.gov/cellminer/home.do http://oncoppi.emory.edu/index.php?navigation=home&location=home https://www.tnmplot.com/ https://cansarblack.icr.ac.uk/ https://portals.broadinstitute.org/ctrp/?page=#ctd2BodyHome https://depmap.org/repurposing/
**XVII. Drug Repurposing Servers**
➢PROMISCUOUS 2.0 ➢repoDB ➢The Drug Repurposing Hub ➢ReDO-DB		https://bioinformatics.charite.de/promiscuous2/ http://apps.chiragjpgroup.org/repoDB/ https://clue.io/repurposing-app https://www.anticancerfund.org/en/redo-db

## Drugs Repurposed for Treatment of NSCLC

Various drugs have been approved by the US FDA for NSCLC targeted therapy. These treatments have been focused on the BRAF and KRAS mutations, oncogenic EGFR mutations, HER2/ERBB2 mutations, HGFR/MET alterations, ROS1 (pro-to-oncogene receptor tyrosine kinase) and ALK fusion for the past few years (68).

The number of therapeutic improvements has been lower than projected despite advancements in treatment techniques and overall knowledge of cancer heterogeneity. Significant investments in drug development have been prompted by the limited success of current medicines in advanced phases. The desire for more effective anti-cancer treatments has generated a surge in drug repurposing research. The target-based approach for drug repurposing has been a potent technology that is integrated with high-quality, real-time drug testing for a protein or biomarker. By pulling documents from journals like PubMed central and Elsevier, we were able to compile the details of repurposing candidates, specifically for the treatment of NSCLC, described in **Table 2**.

**Table 2:** Details of drugs reported to be repurposed for NSCLC therapy.

Sl.no.	Drug	Original indication	Mechanism of new indication for cancer	Remarks
1.	Dilsulfiram (69)	Anti-alcoholism drug	Elimination of cancer stem cells (CSCs) and reduction of chemoresistance in cancer cell lines that are resistant to chemotherapy.	Clinical trials phase III (NCT00312819)
2.	Nelfinavir (70)	HIV-1 protease inhibitor	In this disease, nelfinavir may improve the efficacy of routine chemoradiotherapy. This drug Inhibits PI3K/AKT signaling and sensitizes tumor cells to killing by ionizing radiation.	Clinical trials phase II (NCT00589056)
3.	Ganetespib (71, 72)	Heat Shock Protein 90 inhibitor	With a response rate of 50% in individuals with ALK-rearranged illness, ganetespib exhibited promising single-agent efficacy.	Clinical trials phase II-(NCT01031225)
4.	Dasatinib and Osimertinib (73)	TKI for chronic myeloid leukemia (CML) + NSCLC kinase inhibitor	Combination of TKI and a Src inhibitor are synergistic in Cripto-1 overexpressing tumors in the laboratory.	Clinical trials phase II (NCT02954523)
5.	Verapamil (74)	Calcium channel blocker	Chemo resistant lung cancer cells are efficiently sensitized to death by autophagy burst and apoptosis by Verapamil with Docetaxel/Vincristine.	Randomized Clinical study
6.	Hydroxychloroquine + chemotherapy (75, 76)	Anti-malarial drug	In advanced NSCLC, adding hydroxychloroquine is safe and tolerated, and autophagy inhibition may alleviate chemotherapy resistance.	Clinical trials phase II (NCT01649947)
7.	Artemisinin and its derivatives	Anti-malarial drug	In A549 and H1299 cells, cell proliferation was inhibited by artesunate, artemisinin and dihydroartemisinin *via* cell cycle arrest in the G1 phase. Also, apoptosis was induced by dihydroartemisinin in A549 cells (77, 78). In ABT-263 NSCLC cells with EGFR or Ras mutations, dihydroartemisinin inhibited STAT3 phosphorylation and activation, lowering surviving levels (79).	*In vitro* and *in vivo*
8.	Ibuprofen + Cisplatin (80)	Non-steroidal anti-inflammatory drug	Decreased Heat shock protein 70 (Hsp70) expression and sensitized A549 cells originating from lung adenocarcinoma to cisplatin, accompanied by an increase in the mitochondrial apoptotic cascade. In lung adenocarcinoma cells, ibuprofen enhanced the antitumor effects of cisplatin through a mechanism involving Hsp70 suppression.	*In vitro* and *in vivo*
9.	Metformin + Nivolumab (81)	Anti-diabetic drug + Immunotherapy	In NSCLC cells, metformin triggered apoptosis and significantly reduced the expression of c-FLIP_L_.	Clinical trials phase II (NCT03048500)
10.	Minocyclin (82)	Antibiotic	Reduction of adverse effects in NSCLC patients treated with chemoradiation.	Clinical trials phase II (NCT01636934)
11.	Itraconazole (83)	Antifungal drug	Exhibits concentration-dependent early antivascular, metabolic, and antitumor effects in NSCLC patients.	Clinical trials phase II (NCT03664115)
12.	Pirfenidone + Chemotherapy (84)	Anti-fibrotic drug	In NSCLC cells (A549 and H157 cells), a combination of cisplatin and pirfenidone causes enhanced apoptosis and synergistic cell death.	Clinical trials phase I (NCT03177291)
13.	Sertraline + Erlotinib (85)	Antidepressant drug + TKI	In an orthotopic NSCLC mouse model, this combination inhibits tumour growth and extends mice longevity.	*In vitro* and *in vivo*
14.	Quinacrine + Erlotinib (3, 86)	Antimalarial drug+ TKI	Quinacrine inhibits the FACT (facilitates chromatin transcription) complex, which may play a role in resistance to TKI.	Clinical trials phase I (NCT01839955)
15.	Romidepsin + Erlotinib (87)	Anticancer drugs	Erlotinib is more effective when used with romidepsin. It inhibits the signaling pathways of Ras and MAPK, intracellular mediators that may lead to EGFR TKI resistance.	Clinical trials phase I (NCT01302808)
16.	Itraconazole + Pemetrexed (3, 88)	Oral antifungal drug + Chemotherapy	In numerous primary xenograft lung cancer models, ittraconazole shows substantial anti-angiogenic activity and improves the efficiency of cytotoxic treatment.	Clinical trials phase I (NCT00769600)
17.	Nitroglycerin + Vinorelbine + Cisplatin (89)	A drug to treat angina + Chemotherapeutics	Improved overall survival of patients with untreated stage IIIB/IV non-squamous cell lung cancer	Phase II randomized trial
18.	Gefitinib+ Bevacizumab (90)	EGFR-TKI+ Antiangiogenic drug	First-line therapy in patients with EGFR mutant NSCLC with tolerable toxicity.	Clinical trials phase II (NCT04425187)

## Conclusion and Discussion

In this review, we presented an overview of drug repositioning for anti-cancer applications, with a focus on target-based non-cancer drug repurposing. Targeting the Ras/Raf/MEK/ERK is a promising and alternative method in NSCLC treatment, according to the information presented in this review. MAPK signaling pathways are a vital aspect of NSCLC and have aided in the advancement of therapies for this carcinoma. Learning more about this signaling is critical because of its significant functions in carcinogenesis and wide spectrum of crosstalk with major tumor-promoting signaling pathways.

Drug repurposing has the potential to alleviate the present drug shortage. There is an advantage to the use of many *in silico* techniques over individual methods, especially when dealing with massive amounts of data. We have provided the vital steps involved in an *in silico* drug repurposing pipeline to assist in guiding the pipeline and increase the success rate in this field. Furthermore, the utilization of non-oncology drugs has the potential to speed up the process of drug repurposing. Based on a broad understanding of these principles and related investigations on the current strategy over the last decade, we defined and evaluated previous non-oncology drugs as potential candidates for therapeutic repurposing. Alternative strategies, such as drug combination therapy, should be examined because they may have a better clinical success rate. Drug repurposing and drug combinations are two common methods for improving cancer treatment by lowering its toxicological profile and increasing its efficacy. Drug combination therapy frequently target various processes that contribute to cancer, such as downstream off-target, parallel pathways, or compensatory signaling. Future combinatorial drugs (either immune-therapies or targeted therapies) and an improved understanding on molecular biomarkers can lead to a cure (62).

Although drug repurposing lowers the time and expenses of drug development, the benefits are confined to a specific process between preclinical and Phase II studies. Another issue to consider is the protection of the repositioned drugs’ intellectual property (IP), particularly for those medications that are no longer under patent. It’s also unclear whether new anticancer indications require medication dosages, formulations, or administration routes that are identical to those utilized for the original indication. Extensive research on the safety of repurposed drugs with various doses and populations can help overcome these obstacles. Anticancer drugs have been effectively re-purposed from a variety of structurally and functionally varied drugs through a variety of mechanisms. Most non-cancer medications have few or tolerable side effects in humans, so repurposing non-cancer drugs for NSCLC therapy will be a promising strategy for the future. Further studies should focus on targeting the mutations of the Ras/Raf/MEK/ERK pathway.

Together, we’ve made significant strides ahead in illustrating the repurposing potential for NSCLC by evaluating current sources from both research and clinical studies to find new uses for approved or unsuccessful medications. We anticipate that medicinal chemists will consider this review article incredibly useful in the future to generate new repurposed anti-cancer drugs from currently licensed non-cancer drugs. The hope is to significantly boost lung cancer cure rates, and the true challenge will be incorporating these drugs into the therapy of patients affected with NSCLC.

## Author Contributions

All authors listed have made a substantial, direct, and intellectual contribution to the work, and approved it for publication.

## Conflict of Interest

The authors declare that the research was conducted in the absence of any commercial or financial relationships that could be construed as a potential conflict of interest.

## Publisher’s Note

All claims expressed in this article are solely those of the authors and do not necessarily represent those of their affiliated organizations, or those of the publisher, the editors and the reviewers. Any product that may be evaluated in this article, or claim that may be made by its manufacturer, is not guaranteed or endorsed by the publisher.

## Abbreviations

NSCLC, Non-Small Cell Lung Cancer; MAPK, Mitogen-Activated Protein Kinase; NIH, National Institutes of Health; FDA, Food and Drug Administration; EMA, European Medicines Agency; EGFR, Epidermal Growth Factor Receptor; PI3K, Phosphatidylinositol 3-Kinases; JNK, C-Jun N-terminal Kinase; KEGG, Kyoto Encyclopaedia of Genes and Genomes; EML4, Echinoderm Microtubule-Associated Protein-like 4; ALK, Anaplastic Lymphoma Kinase; RTK, Receptor Tyrosine Kinases; GF, Growth Factor; EGF, Epidermal Growth Factor; SHP2, Src homology region 2-containing protein tyrosine phosphatase 2; GRB2, Growth Factor Receptor Bound Protein 2); GDP, Guanosine Diphosphate; GTP, Guanosine Triphosphate; SOS, Son of Sevenless; HRas, Harvey Rat Sarcoma Viral Oncogene Homolog; NRas, Neuroblastoma Rat Sarcoma Viral Oncogene Homolog; KRas, Kirsten Rat Sarcoma Viral Oncogene Homolog; MEK, Mitogen-Activated Protein Kinase Kinase; ERK, Extracellular Signal-Regulated Kinase; HER2, Human Epidermal Growth Factor Receptor 2; HGFR, Hepatocyte Growth Factor Receptor.
