# Corrigendum: Everything Old Is New Again: Drug Repurposing Approach for Non-Small Cell Lung Cancer Targeting MAPK Signaling Pathway

**DOI:** 10.3389/fonc.2021.822865

**Published:** 2021-12-20

**Authors:** Anisha S. Jain, Ashwini Prasad, Sushma Pradeep, Chandan Dharmashekar, Raghu Ram Achar, Ekaterina Silina, Victor Stupin, Raghavendra G. Amachawadi, Shashanka K. Prasad, R. Pruthvish, Asad Syed, Chandan Shivamallu, Shiva Prasad Kollur

**Affiliations:** ^1^ Department of Microbiology, School of Life Sciences, JSS Academy of Higher Education and Research, Mysuru, India; ^2^ Department of Biotechnology and Bioinformatics, School of Life Sciences, JSS Academy of Higher Education and Research, Mysuru, India; ^3^ Division of Biochemistry, School of Life Sciences, JSS Academy of Higher Education and Research, Mysuru, India; ^4^ Department of Human Pathology, I.M. Sechenov First Moscow State Medical University (Sechenov University), Moscow, Russia; ^5^ Department of Hospital Surgery, N.I. Pirogov Russian National Research Medical University (RNRMU), Moscow, Russia; ^6^ Department of Clinical Sciences, College of Veterinary Medicine, Kansas State University, Manhattan, KS, United States; ^7^ Department of Biotechnology, Acharya Institute of Technology, Bengaluru, India; ^8^ Department of Botany and Microbiology, College of Science, King Saud University, Riyadh, Saudi Arabia; ^9^ Department of Sciences, Amrita School of Arts and Sciences, Amrita Vishwa Vidyapeetham, Mysuru, India

**Keywords:** non-small cell lung cancer, drug repurposing/repositioning, MAPK, targeted therapy, inhibitors

An author name was incorrectly spelled as “**Silina Ekaterina**”. The correct spelling is “**Ekaterina Silina**”.

An author name was incorrectly spelled as “**Stupin Victor**”. The correct spelling is “**Victor Stupin**”.

The authors apologize for these errors and state that this does not change the scientific conclusions of the article in any way. The original article has been updated.

## Publisher’s Note

All claims expressed in this article are solely those of the authors and do not necessarily represent those of their affiliated organizations, or those of the publisher, the editors and the reviewers. Any product that may be evaluated in this article, or claim that may be made by its manufacturer, is not guaranteed or endorsed by the publisher.

